# Insight into vital role of autophagy in sustaining biological control potential of fungal pathogens against pest insects and nematodes

**DOI:** 10.1080/21505594.2018.1518089

**Published:** 2018-09-27

**Authors:** Sheng-Hua Ying, Ming-Guang Feng

**Affiliations:** Institute of Microbiology, College of Life Sciences, Zhejiang University, Hangzhou, China

**Keywords:** Entomopathogenic fungi, nematophagous fungi, autophagic events, asexual development, stress response, virulence, host-pathogen interactions

## Abstract

Autophagy is a conserved self-degradation mechanism that governs a large array of cellular processes in filamentous fungi. Filamentous insect and nematode mycopthogens function in the natural control of host populations and have been widely applied for biological control of insect and nematode pests. Entomopathogenic and nematophagous fungi have conserved “core” autophagy machineries that are analogous to those found in yeast but also feature several proteins involved in specific aspects of the autophagic pathways. Here, we review the functions of autophagy in protecting fungal cells from starvation and stress cues and sustaining cell differentiation, asexual development and virulence. An emphasis is placed upon the regulatory mechanisms involved in autophagic and non-autophagic roles of some autophagy-related genes. Methods used for monitoring conserved or specific autophagic events in fungal pathogens are also discussed.

## Introduction

Autophagy is an evolutionally conserved degradation process much beyond a simply starvation-responsive process considered previously in eukaryotic cells [1]. This self-degradation cellular process has been intensively studied in model yeast and occurs selectively or nonselectively in the form of microautophagy or macroautophagy. Microautophagy takes place via direct uptake of cytoplasm or organelles surrounded by invaginated vacuolar membranes. Nonselective macroautophagy involves random engulfment of cytoplasm and organelles by autophagsomes that appear in the vacuoles containing the contents to be degraded and recycled, contrasting to selective macroautophagy that enables to degrade specific organelles, such as mitochondria, peroxisomes and ribosomes, for removal of redundant or impaired organelles [2]. Autophagy is mediated by the cytoplasm-to-vacuole targeting (Cvt) pathway that is responsible for specific sorting of proteins to vacuoles [3]. Despite conserved features, autophagic proteins are functionally differentiated among fungi [4]. Filamentous fungi are highly divergent in morphology and lifestyle [5], and hence the roles of autophagic processes in their adaptation to host and environment may differ from one lineage to another [6].

Filamentous entomopathogenic and nematophagous fungi play important roles in the natural control of host populations and have been widely applied for biological control of pest insects and nematodes [7,8]. As classic insect mycopathogens, *Beauveria bassiana* and *Metarhizium* spp. are a large source of global mycoinsecticides and mycoacricides as alternatives to chemical pesticides [9,10]. Fungal conidia adhere to insect cuticle, where they geminate to infect host through cuticular penetration for entry into host hemocoel [11]. The success of fungal infection is followed by transition of penetrating hyphae into hyphal bodies (namely unicellular blastospores), a process called dimorphic transition that facilitates intrahemocoel proliferation of fungal cells by yeast-like budding until host mummification to death [12–14]. Upon host death, hyphal bodies become septate hyphae that penetrate the host cuticle again for outgrowth and ultimate conidiation on cadaver surfaces for a new infection cycle [15,16]. Nematophagous fungi can be divided into nematode-trapping, egg-parasitic, endoparasitic and toxin-producing groups [17]. The mycelia of the first two groups can form traps to capture namatodes and invade host eggs by the actions of mechanical forces and extracellular hydrolytic enzymes [18], respectively. The infection cycle of an entomopathogenic or nematophagous fungus comprises a wide array of cellular processes and events that are closely linked to autophagy [19,20]. This mini-review aims to update the understanding of autophagic events that are genetically regulated in insect and nematode mycopathgens and associated with their phenotypes crucial for biological control potential, including vegetative growth, cell differentiation, asexual or sexual development, host infection and virulence.

## Overview of autophagy-related proteins in insect and nematode mycopathogens

**Figure 1.**
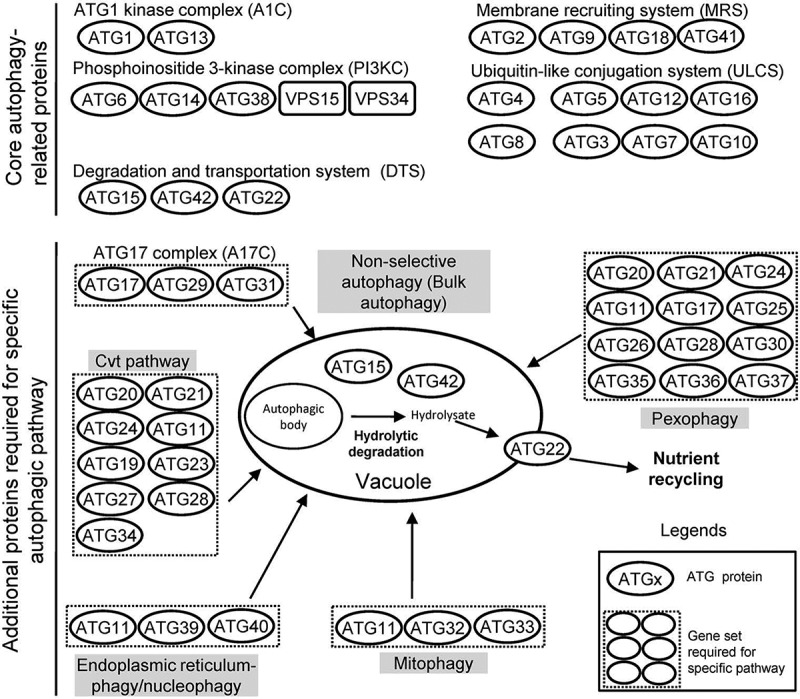
Autophagy-related (ATG) genes functioning in unicellular fungi. Forty-two ATG genes plus Vps14 and Vps34 involved in autophagy pathway of yeast species are sorted into two groups, of which one works in the “core” autophagy machinery and another participates in various specific pathways [24].

The yeasts *Saccharomyces cerevisiae, Komagataella pastoris* (formerly *Pichia pastoris*) and *K. phaffii* are model species used in autophagic studies. Up to 42 genes have been found encoding autophagy-related proteins (ATGs) and mostly characterized in the yeasts, as summarized in Table 1. Among those, 18 are considered as core genes indispensable for autophagic processes while other 24 are involved into the induction of specific autophagic pathway or selective autophagy [21–23]. The core ATG genes are obligatory for all autophagy-related processes and fall into five functional groups, including ATG1 kinase complex (A1C), membrane recruiting system (MRS), phosphoinositide 3-kinase complex (PI3KC), ubiquitin-like conjugation system (ULCS), and degradation and transportation system (DTS). As illustrated in Figure 1, autophagic behavior is induced by A1C and PI3KC complexes through formation of preautophagosomal structures, followed by vesicle formation and expansion that rely upon ULCS during autophagosome maturation, hydrolyzation and recycling of all engulfed proteins and organelles by DTS in vacuoles [24], and a requirement of MRS for phagophore membrane expansion and vesicle completion [23].

**Table 1.** Autophagy-related proteins (ATGs) found in the NCBI protein databases of yeasts, fungal entomopathogens and nematophagous fungi.

ATG	NCBI accession codes*																	
	Hp	Kp	Kph	Sc	Aal	Aap	Bba	Bbr	Cm	If	Ll	Mac	Man	Mr	Nr	Sp	Aol	Hm
1	ESW98768	ANZ75424	CAY69285	P53104	OAA32035	KZZ98101	**EJP64020**	OAA47468	ATY62103	OAA71303	OAA80090	EFY88904	KJK76758	**EFZ00905**	OAA44768	OAA58087	EGX50159	KJZ72849
2	ESW96436	ANZ73551	CAY67230	P53855	OAA33259	KZZ92259	EJP62566	OAA39029	ATY66591	OAA53106	OAA80419	EFY85935	KJK76344	EFZ01946	OAA39436	OAA59125	EGX44288	KJZ72057
3	ESW97715	ANZ76765	CAY70737	P40344	KZZ95324	KZZ96434	EJP63325	OAA38715	ATY61762	OAA60125	OAA75012	EFY88596	KJK75521	EFZ01113	OAA44978	OAA63351	EGX49425	KJZ79335
4	ESW98784	ANZ76419	CAY68374	P53867	KZZ93420	KZZ90947	EJP61110	OAA34578	ATY64819	OAA73766	OAA81896	EFY93633	KJK84136	**EFY99546**	OAA49518	OAA54482	EGX47408	KJZ70398
5	ESW99543	ANZ77851	CAY71712	Q12380	OAA33555	KZZ87486	**EJP62801**	OAA52240	ATY61495	OAA60740	OAA76966	EFY90145	EFY90145	EXU96089	OAA42093	OAA62418	EGX50306	KJZ79622
6	ESW98526	ANZ77485	CAY68819	Q02948	KZZ92081	KZZ91922	EJP69800	OAA40746	ATY65156	OAA68923	OAA81116	EFY86689	KJK79721	KHO11008	OAA46931	OAA68176	EGX49628	KJZ77316
7	ESW98216	ANZ74180	CAY68151	P38862	OAA33422	KZZ95791	EJP62461	OAA51370	ATY66145	OAA63006	OAA70982	EFY91403	KJK76449	EFZ01845	OAA42261	OAA68507	EGX45886	KJZ73174
8	ESW99851	ANZ77907	CAY71966	P38182	OAA33691	KZZ97062	**EJP69267**	OAA37148	ATY67476	OAA58875	OAA76358	EFY85199	KJK82117	**EFZ01445**	OAA46238	OAA55906	**EGX52603**	KJZ72025
9	ESW96528	ANZ77553	CAY71765	Q12142	KZZ93426	KZZ90329	EJP61034	OAA34570	ATY64768	OAA73774	OAA81888	EFY93626	KJK84144	EFY99555	OAA49525	OAA54510	EGX51301	KJZ70407
10	ESW98616	ANZ75463	CAY70737	Q07879	KZZ97664	–	EJP70966	OAA45929	EGX89933	OAA73685	OAA81814	EFY90956	KJK73827	OAA48754	OAA48754	OAA59012	EGX50380	KJZ78386
11	ESX02153	ANZ76715	CAY68411	Q12527	OAA32739	KZZ88894	EJP66943	OAA48105	ATY61095	OAA67574	OAA77977	EFY90855	KJK78949	EFZ00535	OAA42617	OAA53787	EGX54268	KJZ73499
12	ESW96997	ANZ74286	CAY70406	P38316	KZZ96701	KZZ95708	EJP64666	OAA46448	ATY67028	OAA63828	OAA74290	EFY91312	KJK81831	EFZ02238	OAA51680	OAA62640	EGX43872	KJZ75788
13	ESW98330	ANZ75343	CAY69477	Q06628	KZZ91450	KZZ87338	EJP67952	OAA42381	EGX93111	OAA70524	OAA79351	EFY90437	KJK83836	EFZ02694	OAA50594	OAA68682	EGX43338	KJZ73196
14	–	–	–	P38270	–	–	–	–	–	–	–	–	–	–	–	–	–	–
15	ESW99625	ANZ73852	CAY67386	P25641	KZZ90734	KZZ90664	EJP62335	OAA38950	ATY58944	OAA58221	OAA80311	EFY87240	KJK79485	**EFZ03432**	OAA34412	OAA64287	EGX46578	KJZ73386
16	ESW98669	ANZ75755	CAY71305	Q03818	KZZ93856	–	EJP66477	OAA49178	ATY61736	OAA64011	OAA79801	–	KJK79436	EFY95610	OAA38355	OAA67064	EGX51377	KJZ74754
17	ESX00887	ANZ76024	CAY69318	Q06410	KZZ91807	KZZ87046	EJP62668	OAA46696	ATY58611	OAA53674	OAA71841	EFY87591	KJK78562	EFY97482	OAA50385	OAA56219	EGX51841	KJZ76468
18	ESX02211	ANZ76366	CAY70991	P43601	KZZ94876	KZZ89532	EJP61015	OAA36986	ATY67510	OAA58836	OAA76320	EFY84843	KJK83381	EFY99242	OAA44358	OAA59674	EGX53695	KJZ78877
19	–	–	–	P35193	–	–	–	–	–	–	–		–	–	–	–	–	–
20	ESW96614	ANZ76112	CAY69906	Q07528	OAA33120	KZZ95709	EJP66835	OAA48003	ATY60813	OAA74127	OAA74633	EFY90783	KJK77643	EFY98676	OAA43098	OAA53836	EGX43058	KJZ78961
21	ESX00158	ANZ76928	CAY71077	Q02887	OAA32982	KZZ92688	EJP65675	OAA52473	EGX91053	OAA54904	OAA79214	EFY89511	KJK75180	EFY94238	OAA36058	OAA60246	EGX45948	KJZ75213
22a	ESW98143	ANZ73516	CAY67729	P25568	KZZ89441	KZZ90658	EJP69073	OAA42939	ATY62792	OAA64972	OAA79970	EFY89712	KJK77771	EFY97213	OAA45553	OAA53950	EGX47846	KJZ76598
22b	–	–	–	–	KZZ96617	KZZ87966	EJP65688	OAA52486	ATY58739	OAA54918	OAA79201	EFY93887	KJK77427	EFY98889	OAA40064	–	–	KJZ75201
22c	–	–	–	–	–	–	EJP65315	OAA52574	ATY66541	OAA59477	OAA74415	EFY85468	KJK75171	EFY96161	OAA38895	–	–	–
22d	–	–	–	–	–	–	EJP61453	–	–	–	–	–	–	–	–	–	–	–
23	–	–	–	Q06671	–	–	–	–	–	–	–	–	–	–	–	–	–	–
24	ESW97141	ANZ75401	CAY69011	P47057	OAA32216	KZZ95709	EJP61354	OAA47182	ATY61719	OAA71568	OAA64158	EFY91127	KJK79963	EFY99206	OAA40571	OAA63756	EGX43379	KJZ78598
25	ESW97416	–	–	–	–	–	–	–	–	–	–	–	–	–	–	–	–	–
26	ESW96191	ANZ76118	CAY71393	Q06321	KZZ90874	KZZ92672	EJP71037	OAA45995	ATY62412	OAA73722	OAA81945	EFY86996	KJK79229	EFY97936	OAA36440	OAA59786	EGX51249	KJZ73456
27	ESW97460	ANZ75892	CAY69817	P46989	KZZ98281	KZZ88672	EJP63614	OAA44065	EGX88907	OAA52851	OAA76046	EFY88154	KJK81470	EFZ02138	OAA41305	OAA57639	EGX46770	KJZ73136
28	ESW99680	ANZ74931	CAY69233	–	KZZ89898	KZZ88029	EJP70908	OAA45872	EGX91880	OAA73420	OAA82283	EFY91502	KJK77990	EFY97624	OAA52097	OAA62786	EGX54013	KJZ75402
29	–	–	–	Q12092	KZZ98964	KZZ91602	EJP65671	OAA52469	EGX91048	OAA54900	OAA79218	EFY87305	KJK75147	EFY96138	OAA40057	OAA53661	EGX48917	KJZ75209
30	ESX03003	ANZ76622	CAY70917	–	–	–	–	–	–	–	–	–	–	–	–	–	–	–
31	–	–	–	Q12421	–	–	–	–	–	–	–	–	–	–	–	–	–	–
32	–	–	–	P40458	–	–	–	–	–	–	–	–	–	–	–	–	–	–
33	ESW96153	–	–	Q06485	OAA33167	KZZ86925	MH427003	OAA37283	EGX92109	OAA73257	OAA82461	EFY90900	KJK79096	EFZ00391	OAA48699	OAA65672	EGX49467	KJZ78329
34	–	–	–	Q12292	–	–	–	–	–	–	–	–	–	–	–	–	–	–
35	ESW99702	ANZ73929	CAY67399	Q06834	OAA33190	KZZ89124	EJP70174	OAA49997	ATY67046	OAA67449	OAA78001	EFY87220	KJK77556	EFY98763	OAA38060	OAA54133	EGX52149	KJZ78899
36	–	–	–	P46983	–	–	–	–	–	–	–	–	–	–	–	–	–	–
37	ESW98758	ANZ77961	CAY71862	–	KZZ98481	KZZ98004	EJP65590	OAA52386	ATY66944	OAA59434	OAA74461	EFY86719	KJK80967	EFY95727	OAA35690	OAA64419	EGX43362	KJZ80366
38	–	–	–	Q05789	–	–	–	–	–	–	–	–	–	–	–	–	–	–
39	–	–	–	Q06159	–	–	–	–	–	–	–	–	–	–	–	–	–	–
40	–	–	–	Q99325	–	–	–	–	–	–	–	–	–	–	–	–	–	–
41	–	–	–	Q12048	–	–	–	–	–	–	–		–	–	–	–	–	–
42	ESX02688	ANZ76773	CAY70682	P38109	OAA33069	KZZ88266	EJP62646	OAA46680	EGX91700	OAA53688	OAA71860	EFY87773	KJK77692	EFY98627	OAA43147	OAA58390	EGX53766	KJZ79011

* Found in the NCBI protein databases of four yeasts (Hp: *Hansenula polymorpha* DL1; Kp: *Komagataella pastoris* NRRL Y-1603; Kph: *K. phaffii* GS115; Sc: *S. cerevisiae* S288C), 12 entomopathogenic fungi (Aal: *Aschersonia aleyrodis* RCEF 2490; Aap: *Ascosphaera apis* ARSEF 7405; Bba: *Beauveria bassiana* ARSEF 2860; Bbr: *B. brongniartii* RCEF 3172; Cm: *Cordyceps militaris* CM01; If: *Isaria fumosorosea* ARSEF 2679; Ll: *Lecanicillium lecanii* RCEF 1005; Mac: *Metarhizium acridum* CQMa102; Man: *M. anisopliae* BRIP 53293; Mr: *M. robertsii* ARSEF 23; Nr, *Nomuraea rileyi* RCEF 4871; Sp: *Sporothrix insectorum* RCEF 264), and two nematophagous fungi (Aol: *A. oligospora* ATCC 24927; Hm: *Hirsutella minnesotensis* 3608). **Red** items are the ATGs that have been characterized in insect and nematode mycopathogens.

In insect and nematode mycopathogens, the ATG genes involved in different autophagic processes are not always identical with the yeast counterparts, and only those associated with A1C complex are completely conserved in fungi (Table 1). For instance, such mycopathogens lack not only ATG41 that interacts with ATG9 and participates in yeast autophagosome biogenesis [22] but also ATG14 and ATG38 that are associated with the yeast PI3KC required for vesicle formation and maturation [23]. ULCS is required for ATG8 activation and involved in two conjugation pathways. One pathway consists of the protease ATG4, the E1-like enzyme ATG7 and the E2-like enzyme ATG3 while another pathway comprises ATG7 and the E2-like enzyme ATG10 [25]. Interestingly, the yeast ATG10 homolog exists in some filamentous fungi [26] but is absent in *Ascosphaera apis* [27], suggesting less conserved structure or too low sequence identity for ATG10 to be located in the honeybee mycopathogen by BLAST search. ATG22 is a permease that uniquely transports degraded products from vacuole to cytosol in most yeast species [24,26]. In contrast, most of insect and nematode mycopathogens have more transporters homologous to the yeast ATG22. *B. bassiana* even possesses four ATG22 homologs, of which two (EJP69073 and EJP65688) are transcriptionally expressed during cell proliferation in host hemocoel [28] and another (EJP65315) is involved in fungal pathogenicity, which was reduced by its insertional mutagenesis [29]. This suggests a possibility for some filamentous entomopathogens to have evolved a strategy of utilizing multiple autophagy-related transporters at different stages of infection cycle.

Filamentous fungal ATG proteins involved in specific autophagy pathway exhibit a low degree of conservation [26]. During nonselective macroautophagy induced by starvation, A1C associates with the ATG17 complex (A17C) consisting of ATG17, ATG29 and ATG31 in *S. cerevisiae* [30]. Of those, ATG31 seems to exist only in *S. cerevisiae* since its homolog is absent in filamentous and other yeast species, such as *Hansenula polymorpha, K. pastoris* and *K. phaffii*. This implicates that a novel mechanism might exist in bulk autophagy of the species other than the budding yeast. In addition, selective autophagy required for cellular homeostasis includes mitophagy, pexophagy, ribophagy, reticulophagy and the Cvt pathway [31]. In selective degradation processes, cargo must be recognized by a receptor and forms a cargo-receptor complex (CRC). ATG11 acts as an essential scaffold protein that mediates the CRC interaction with the core proteins essential for autophagosome formation [32] and is highly conserved in yeasts and filamentous fungi [26]. ATG11 acts as a conserved adaptor which interacts with specific receptors in various pathways [23]. In the budding yeast, aminopeptidase I (Ape1) is translocated into vacuoles via the Cvt pathway, and ATG19 functions as a receptor between Ape1 and ATG11 [23]. In spite of weakly conserved ATG19-B proteins in some yeasts [26], ATG19 is absent in insect and nematode mycopathogens (Table 1), in which it remains unknown whether the Cvt pathway exists and what protein acts as the receptor if it exists. In pexophagy, ATG30 and ATG36 function as the receptor in *K. pastoris* and *S. cerevisiae*, respectively [33,34], but both of them are absent in insect and nematode mycopathogens. ATG32, a protein associated with mitochondrial membrane, acts as a receptor and mediates selective degradation of mitochondria (mitophagy) [35]. ATG39 is anchored in perinuclear endoplasmic reticulum (ER) for initiation of reticulophagy and nucleophagy. ATG40 is localized to cortical and cytoplasmic ER for mediation of specific ER degradation [36]. However, such receptors acting in the selective autophagic processes of yeasts lack homologs in insect and nematode mycopathogens. ATG41 existing only in *S. cerevisiae* has been found to interact with ATG9 and play a role in autophagasome formation [22]. As a newly characterized vacuolar serine carboxypeptidase, ATG42 (Ybr139w) is required for normal vacuole function and the terminal steps of autophagy in *S. cerevisiae* [21] and exist in all examined yeasts and insect/nematode mycopathognes (Table 1), suggesting its highly conserved role in fungi. In *B. bassiana*, selective autophagy is evidently associated with cellular stress response, development and virulence. Loss-of-function mutation of ATG11 in *B. bassiana* has been shown to not completely block autophagic process in vacuoles but to abolish pexophagy and mitophagy during growth *in vitro* and *in vivo* [37] although actual receptors involved in the processes remain unclear.

Overall, many of yeast ATG homologs, particularly those receptors, are distinct or absent in the genomic databases of insect and nematode mycopathogens [7,38–40]. This is likely attributable to their essential or nonessential roles in fungal adaptation to hosts and habitats and/or extremely low identities of their sequences to the counterparts in the model yeasts.

## Monitoring autophagic events in insect and nematode mycopathogens

Interest in unveiling the prominent role of autophagy in the life cycles of insect and nematode mycopathogens is increasing in the postgenomic era. Effective methods have been explored to monitor autophagic events in these mycopathogens. The acidophilic dye monodansyl cadaverine (MDC) has been used as an indicator of acidic autophagosomes to examine whether the stained structures accumulate in the vacuoles of *ΔATG* mutants in *B. bassiana* [41] or at the early stage of mycelial trap formation in the nematode-trapping fungus *Arthrobotrys oligospora* [20]. Due to a high affinity to acid environment, however, MDC is not suitable for the detection of autophagesomes when other acid vesicles exist [42].

Highly conserved ATG8 is localized on the membrane of autophagosomes to be translocated into vacuoles and considered as a molecular marker for autophagic tracking due to its essentiality for preautophagomal structure formation and autophagosome maturation in eukaryotic cells [43,44]. Fluorescence protein-tagged ATG8 fusion proteins have been successfully used to monitor autophagic events in germlings, hyphae, aerial conidia, submerged blastospores and *in vivo* hyphal bodies of *B. bassiana* [20] and in the appressoria formed at the initial stage of infection by *Metarhizium robertsii*, another important insect mycopathogen [45].

Transmission electron microscopy (TEM) is the most effective method that allows for observation of various autophagic structures, such as phagophores, autophagosomes and autophagic bodies [4]. This method has been employed to unveil the absence/presence of autophagic bodies under autophagy-inducing conditions [20] or during asexual development in the absence of important genes in *B. bassiana* [13]. In *M. robertsii*, autophagic bodies in the vacuoles of hyphal cells stressed by starvation are also well visualized via TEM [45]. Recently, dual RNA-seq analysis has been adopted to reveal all possible ATG genes that are expressed during *B. bassiana* propagation within host hemocoel [28]. This suggests that the molecular detection method is highly effective to monitor the activities of all ATG genes in the *in vivo* sample of small size.

## Autophagic events associated with pest control potential of mycopathogens

**Figure 2.**
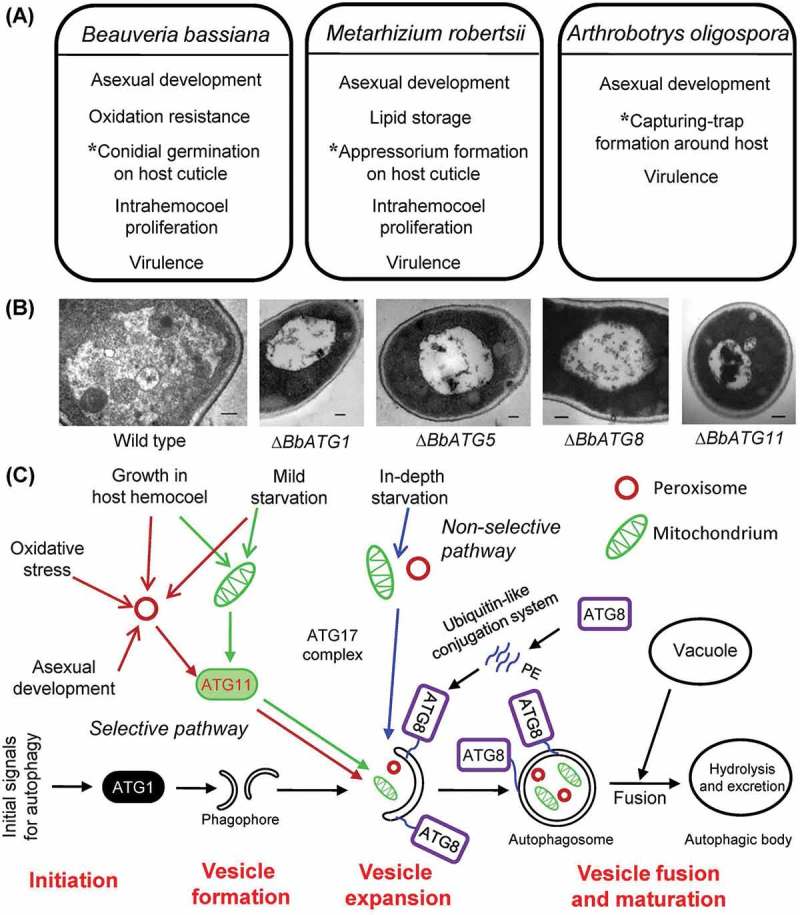
Overview of autophagic events in entomopathogenic and nematophagous fungi. (A) Divergent roles of autophagic events in sustaining the *in vitro* and *in vivo* cellular processes of *B. bassiana, M. robertsii* and *A. oligospora*, three representative mycopathogens that have evolved for adaptation to distinct host spectra and associated habitats and fall into different lineages. Autophagy mediates the asterisked process that is distinct for each of the fungal pathogens to penetrate through the host cuticle after conidial germination. (B) Transmission electronic microscopic images (scale bars: 0.2 μm) for intravacuolar autophagic events altered by singular deletions of *ATG1, ATG5, ATG8* and *ATG11* in *B. bassiana*. (C) Proposed model for autophagy pathways in *B. bassiana*, including starvation-induced or non-selective autophagy, selective autophagy and bulk autophagy. PE: phosphatidylethanolamine.

The biological control potential of a fungal insect or nematode pathogen depends on not only the virulence or pathogenicity as an indicative ability to invade the host but also cell tolerance to environmental adversity and the asexual development that is critical for *in vivo* propagation of fungal cells and efficiency of *in vitro* mass-production [46]. Thus, the fungal potential against pest insects and nematodes is definitely an output of cellular functions and processes that are linked to autophagic events, as illustrated in Figure 2.

### Autophagy in response to nutritional starvation/shift

During conidial germination on scant media or oligotrophic insect cuticle, *B. bassiana* may make use of autophagy to mobilize and recycle intracellular stored nutrients. This role is evidenced with abolishment of autophagic process in the absence of some ATG genes, such as *ATG1, ATG5* and *ATG8* [20,41]. The conidia of these *ΔATG* mutants germinated as well as the wild-type conidia on rich medium but suffered germination defects in response to nutritional starvation on water agar and host cuticle. Additionally, deletion of *ATG11* in *B. bassiana* resulted in a block of selective autophagy during conidial germination on oligotrophic substrata and significant germination defects under nutrient deficient conditions [37]. Similarly, nematode surface is also a nutritionally poor substratum for nematode-trapping fungi [47], in which autophagic process is induced by amino acid starvation [19].

A plenty of fatty acids and lipids are used as carbon sources by insect mycopathogens during their infection to host through cuticular penetration [48]. Upon entry into the host hemocoel, fungal cells need metabolize hemolymph-rich trehalose and other carbohydrates and convert them to glucose for use in intrahemocoel propagation [49]. Pexophagy was first found in the cells of *K. pastoris* grown in an oleic acid-based medium and then shifted into a glucose-based medium [50]. In *B. bassiana*, both pexophagy and mitophagy are evidently involved in cell response to carbon shift [37].

### Autophagy in response to oxidative stress

Autophagy plays an important role in scavenging damaged organelles and proteins in the response of mammal cells to oxidative stress [51]. In *B. bassiana*, ATG1 and ATG8 are functionally different in antioxidant response since total activity of superoxide dismutases (SODs) decreased by 50–70% in absence of *ATG8* but was not affected in absence of *ATG1* [20]. This contrasts to increased resistance to oxidative stress in the absence of either *ATG1* or *ATG8* in *Aspergillus niger* [52]. Similarly, loss-of-function mutations of *ATG1* and *ATG8* in *S. cerevisiae* resulted in enhanced SOD activities [53]. Apparently, ATG1 and ATG8 could be independent of each other in the response of *B. basssiana* to oxidative stress, and mechanistically different from their homologs in the antioxidant response of both mentioned fungi.

Reactive oxygen species (ROS) causing oxidative damage to mitochondria can be scavenged by selective autophagy for mitochondrial homeostasis [54]. Interestingly, ATG11 has been confirmed to function in antioxidant response of *B. bassiana* through pexophagy rather than mitophagy [37] although a mechanism underlying the ATG11-induced pexophagy under oxidative stress remains unclear.

### Autophagy during cell differentiation and development

Autophagy has been widely linked to cell differentiation in many filamentous fungi [55]. In *B. bassiana*, normal autophagy is required for asexual development and morphogenesis. For instance, deletion of *ATG1, ATG5* or *ATG8* has been shown to greatly reduce the yields of aerial conidia as infective propagules or submerged blastospores as an index of *in vivo* dimorphic transition rate [20,41]. Autophagy is also involved in the conidiation of *M. robertsii* [45] or in the formation of mycelial nematode traps by *A. oligospora* [19].

Moreover, some ATG genes may regulate conidiation via different pathways. For example, a conidial protein (BbCP15) is required for conidiation due to the role of its acting as a downstream target of ATG1 instead of ATG8 in *B. bassiana* [20]. ATG5 is linked to conidial morphology in *B. bassiana* due to conidial size enlarged in absence of *ATG5* [41]. These findings indicate distinct roles for some ATG genes in the cell differentiation and development that are associated with the *in vitro* and *in vivo* life cycles of insect and nematode mycopathogens.

### Autophagy associated with host infection and fungal virulence

Fungal virulence is a pleiotropic phenotype linked to an array of cellular processes and events. In *M. robertsii*, ATG8 is essential for the formation of appressoria that initiate cuticular penetration in the course of host infection [45]. Deletion of *ATG1, ATG5* or *ATG8* resulted in blocked autophagy and attenuated virulence in *B. bassiana* [20,41], *M. robertsii* [45] or *A. oligospora* [19]. These studies demonstrate important impacts of autophagy on the virulence of insect and nematode mycopahtogens but are somewhat different from an absolute requirement of autophagy for the pathogenesis of *Magnaporthe grisea*, a phytopathogenic fungus [56]. The limited ATG genes characterized to date indicate a close linkage of normal autophagy with the virulence of entomopathogenic and nematophagous fungi. Therefore, different lineages of insect and nematode mycopathogens are ideal models for exploring diverse mechanisms involved in autophagic linkage to fungal virulence.

## Regulatory network of autophagy in insect and nematode mycopathogens

In eukaryotes, autophagy is a precisely regulated self-degrading process. The target of rapamycin (TOR) pathway is considered to be a main regulator of autophagy and can inhibit autophagy via phosphorylation of ATG13, a regulator of ATG1 complex [57]. The TOR kinase is inactivated by sensing signals from upstream pathways, followed by formation of autophagy-inducing complex [2]. In *B. bassiana*, the ATG1 kinase may induce autophagy in response to starving cues [20]. Transcriptional networks learned from some insect and nematode mycopathogens also play important roles in autophagic processes. In *Sordaria macrospora* (a filamentous ascomycete), a bZIP transcription factor required for vegetative growth and fruiting-body development represses transcriptional expression of *ATG4* and *ATG8* [58]. Fungus-nematode interaction induces the autophagy of *A. oligospora* by amino acid starvation in a manner absolutely depending on transcriptional regulation of GCN4 which activates a set of genes required for amino acid biosynthesis [19]. G-protein receptor 3 is required for transcription of *ATG1* and *ATG2* during the *in vitro* blastospore formation of *B. bassiana* [59], suggesting an involvement of the G-protein pathway in signal transduction during autophagy. An *in vivo* transcriptomic analysis has uncovered that all ATG genes are expressed during *B. bassiana* propagation in host hemocoel, including *ATG4, ATG8* and *ATG10* regulated by alternative splicing [28]. In addition, two core eisosome proteins (Pil1A and Pil1B) simultaneously localized at the periphery of hyphal cells have been shown to play opposite roles in the autophagic regulation of *B. bassiana*, as unveiled by blocked autophagy in absence of *Pil1B*, restored autophagy in absence of *Pil1A* and opposite changes in transcript levels of many ATG genes in the mutant strains [13]. These studies indicate a complicated autophagy-regulatory network that remains poorly understood in insect and nematode mycopathogens.

## Concluding remarks

Autophagic events exert comprehensive effects on the *in vitro* and *in vivo* life cycles of entomopathogenic and nematophagous fungi, in which many ATG genes remain to be functionally explored. The previous studies restricted to several conserved ATG genes have unveiled that their roles in autophagic events are not necessarily similar to those learned from model yeasts or phytopathogenic fungi. We speculate that insect and nematode mycopathogens could have evolved a distinct autophagy-regulatory network that warrants their adaptation to entomopathogenic or nematophagous lifestyle, which could have originated from different evolution histories. In classic insect mycopathogens, for instance, the *Beauveria*/*Cordyceps* lineage is considered to have evolved insect pathogenicity 130 million years earlier than the *Metarhizium* lineage from plant affinity or pathogenicity [38–40,60]. Perhaps for this reason, host spectra differ greatly between *B. bassiana* and *Metarhizium* spp [10]. So do their genetic backgrounds required for adaptation to different host spectra and associated habitats. Due to their high potential for use in pest control programs, it is necessary to functionally characterize the ATG family genes of the representative lineages, elucidate contributions of autophagic events to their potential against pest insects and nematodes, and explore possible mechanisms underlying the events. Future emphasis is expectedly placed upon distinct roles of some ATG genes in sustaining biological control potential of insect and nematode mycopathogens. The new knowledge will facilitate development and application of fungal formulations against target pests.

## References

[1] Ryter SW, Cloonan SM, Choi AMK. Autophagy: a critical regulator of cellular metabolism and homeostasis. Mol Cells. 2013;36(1):7–16. 23708729 10.1007/s10059-013-0140-8 PMC3887921

[2] Reggiori F, Klionsky DJ Autophagic processes in yeast: mechanism, machinery and regulation. Genetics. 2013;194(2):341–361. 23733851 10.1534/genetics.112.149013 PMC3664846

[3] Lynch-Day M, Klionsky DJ The Cvt pathway as a model for selective autophagy. FEBS Lett. 2010;584(7):1359–1366. 20146925 10.1016/j.febslet.2010.02.013 PMC2843786

[4] Pollack JK, Harris SD, Marten MR Autophagy in filamentous fungi. Fungal Genet Biol. 2009;46(1):1–8. 19010432 10.1016/j.fgb.2008.10.010

[5] Klein DA, Paschke MV Filamentous fungi: the indeterminate lifestyle and microbial ecology. Microb Ecol. 2004;47(3):224–235. 15037964 10.1007/s00248-003-1037-4

[6] Voigt O, Pöggeler S Self-eating to grow and kill: autophagy in filamentous ascomycetes. Appl Microbiol Biotechnol. 2013;97(21):9279–9290. 10.1007/s00253-013-5221-2 24077722

[7] Yang J, Wang L, Ji X, et al Genomic and proteomic analyses of the fungus *Arthrobotrys oligospora* provide insights into nematode-trap formation. PLoS Pathog. 2011;7(9):e1002179. 21909256 10.1371/journal.ppat.1002179 PMC3164635

[8] Wang CS, Wang SB Insect pathogenic fungi: genomics, molecular interactions, and genetic improvements. Annu Rev Entomol. 2017;62:73–90. 27860524 10.1146/annurev-ento-031616-035509

[9] de Faria MR, Wraight SP Mycoinsecticides and Mycoacaricides: a comprehensive list with worldwide coverage and international classification of formulation types. Biol Control. 2007;43(3):237256.

[10] Wang CS, Feng MG Advances in fundamental and applied studies in China of fungal biocontrol agents for use against arthropod pests. Biol Control. 2014;68:129–135.

[11] Ortiz-Urquiza A, Keyhani NO Action on the surface: entomopathogenic fungi versus the insect cuticle. Insects. 2013;4(3):357374. 10.3390/insects4030357 PMC4553469 26462424

[12] Wang J, Ying SH, Hu Y, et al Mas5, a homologue of bacterial DnaJ, is indispensable for the host infection and environmental adaptation of a filamentous fungal insect pathogen. Environ Microbiol. 2016;18(3):10371047. 10.1111/1462-2920.13197 26714790

[13] Zhang LB, Tang L, Ying SH, et al Two eisosome proteins play opposite roles in autophagic control and sustain cell integrity, function and pathogenicity in *Beauveria bassiana*. Environ Microbiol. 2017;19(5):2037–2052. 28276124 10.1111/1462-2920.13727

[14] Tong SM, Zhang AX, Guo CT, et al Daylight length-dependent translocation of VIVID photoreceptor in cells and its essential role in conidiation and virulence of *Beauveria bassiana*. Environ Microbiol. 2018;20(1):169–185. 28967173 10.1111/1462-2920.13951

[15] He PH, Dong WX, Chu XL, et al The cellular proteome is affected by a gelsolin (*BbGEL1*) during morphological transitions in aerobic surface versus liquid growth in the entomopathogenic fungus *Beauveria bassiana*. Environ Microbiol. 2016;18(11):4153–4169. 27554994 10.1111/1462-2920.13500

[16] Cai Q, Wang JJ, Fu B, et al Gcn5-dependent histone H3 acetylation and gene activity is required for the asexual development and virulence of *Beauveria bassiana*. Environ Microbiol. 2018;20(4):1484–1497. 29417710 10.1111/1462-2920.14066

[17] Liu X, Xiang M, Che Y The living strategy of nematophagous fungi. Mycoscience. 2009;50(1):20–25.

[18] Li J, Zou C, Xu J, et al Molecular mechanisms of nematode-nematophagous microbe interactions: basis for biological control of plant-parasitic nematodes. Annu Rev Phytopathol. 2015;53:67–95. 25938277 10.1146/annurev-phyto-080614-120336

[19] Chen YL, Gao Y, Zhang KQ, et al Autophagy is required for trap formation in the nematode-trapping fungus *Arthrobotrys oligospora*. Env Microbiol Rep. 2013;5(4):511–517. 23864564 10.1111/1758-2229.12054

[20] Ying SH, Liu J, Chu XL, et al The autophagy-related genes *BbATG1* and *BbATG8* have different functions in differentiation, stress resistance and virulence of mycopathogen *Beauveria bassiana*. Sci Rep. 2016;6:26376. 27197558 10.1038/srep26376 PMC4873834

[21] Parzych KR, Ariosa A, Mari M, et al A newly characterized vacuolar serine carboxypeptidase, Atg42/Ybr139w, is required for normal vacuole function and the terminal steps of autophagy in the yeast *Saccharomyces cerevisiae*. Mol Biol Cell. 2018;29(9):1089–1099. 29514932 10.1091/mbc.E17-08-0516 PMC5921575

[22] Yao Z, Delorme-Axford E, Backues SK, et al Atg41/Icy2 regulates autophagosome formation. Autophagy. 2015;11(12):2288–2299. 26565778 10.1080/15548627.2015.1107692 PMC4835205

[23] Farré J-C, Subramani S Mechanistic insights into selective autophagy pathways: lessons from yeast. Nat Rev Mol Cell Biol. 2016;17(9):537–552. 27381245 10.1038/nrm.2016.74 PMC5549613

[24] Yang Z, Huang J, Geng J, et al Atg22 Recycles amino acids to link the degradative and recycling functions of autophagy. Mol Biol Cell. 2006;17(12):5094–5104. 17021250 10.1091/mbc.E06-06-0479 PMC1679675

[25] Reumann S, Voitsekhovskaja O, Lillo C From signal transduction to autophagy of plant cell organelles: lessons from yeast and mammals and plant-specific features. Protoplasma. 2010;247(3–4):233–256. 20734094 10.1007/s00709-010-0190-0

[26] Meijer WH, van der Klei IJ, Veenhuis M, et al ATG genes involved in non-selective autophagy are conserved from yeast to man, but the selective Cvt and pexophagy pathways also require organism-specific genes. Autophagy. 2007;3(2):106–116. 17204848 10.4161/auto.3595

[27] Cornman RS, Bennett AK, Murray KD, et al Transcriptome analysis of the honey bee fungal pathogen, *Ascosphaera apis*: implications for host pathogenesis. BMC Genomics. 2012;13:285. 22747707 10.1186/1471-2164-13-285 PMC3425160

[28] Dong WX, Ding JL, Gao Y, et al Transcriptomic insights into the alternative splicing-mediated adaptation of the entomopathogenic fungus *Beauveria bassiana* to host niches: autophagy-related gene 8 as an example. Environ Microbiol. 2017;19(10):4126–4139. 28730600 10.1111/1462-2920.13862

[29] Kim S, Lee SJ, Nai YS, et al Characterization of T-DNA insertion mutants with decreased virulence in the entomopathogenic fungus *Beauveria bassiana* JEF-007. Appl Microbiol Biotechnol. 2016;100(20):8889–8900. 27470140 10.1007/s00253-016-7734-y

[30] Kawamata T, Kamada Y, Kabeya Y, et al Organization of the pre-autophagosomal structure responsible for autophagosome formation. Mol Biol Cell. 2008;19(5):2039–2050. 18287526 10.1091/mbc.E07-10-1048 PMC2366851

[31] Kraft C, Reggiori F, Peter M Selective types of autophagy in yeast. BBA-Mol Cell Res. 2009;1793(9):404–1412. 10.1016/j.bbamcr.2009.02.006 19264099

[32] Suzuki K Selective autophagy in budding yeast. Cell Death Differ. 2013;20(1):43–48. 22705847 10.1038/cdd.2012.73 PMC3524628

[33] Farré JC, Manjithaya R, Mathewson RD, et al PpAtg30 tags peroxisomes for turnoer by selective autophagy. Dev Cell. 2008;14(3):365–376. 18331717 10.1016/j.devcel.2007.12.011 PMC3763908

[34] Motley AM, Nuttall JM, Hettema EH Pex-3anchored Atg36 tags peroxisomes for degradation in *Saccharomyces cerevisiae*. EMBO J. 2012;31(13):2852–2868. 22643220 10.1038/emboj.2012.151 PMC3395097

[35] Kondo-Okamoto N, Noda NN, Suzuki SW, et al Autophagy-related protein 32 acts as autophagic degron and directly initiate mitophagy. J Biol Chem. 2012;287(13):10631–10638. 22308029 10.1074/jbc.M111.299917 PMC3323008

[36] Mochida K, Oikawa Y, Kimura Y, et al Receptor-mediated selective autophagy degrades the endoplasmic reticulum and the nucleus. Nature. 2015;522(7556):359–362. 26040717 10.1038/nature14506

[37] Ding JL, Peng YJ, Chu XL, et al Autophagy-related gene *BbATG11* is indispensable for pexophagy and mitophagy, and contributes to stress response, conidiation and virulence in the insect mycopathogen *Beauveria bassiana*. Environ Microbiol. 2018 DOI:10.1111/1462-2920.14329 30058280

[38] Gao Q, Jin K, Ying SH, et al Genome sequencing and comparative transcriptomics of the model entomopathogenic fungi *Metarhizium anisopliae* and *M. acridum*. PLoS Genet. 2011;7(1):e1001264. 21253567 10.1371/journal.pgen.1001264 PMC3017113

[39] Zheng P, Xia Y, Xiao G, et al Genome sequence of the insect pathogenic fungus *Cordyceps militaris*, a valued traditional Chinese medicine. Genome Biol. 2011;12(1):R116. 22112802 10.1186/gb-2011-12-11-r116 PMC3334602

[40] Xiao G, Ying SH, Zheng P, et al Genomic perspectives on the evolution of fungal entomopathogenicity in *Beauveria bassiana*. Sci Rep. 2012;2:483. 22761991 10.1038/srep00483 PMC3387728

[41] Zhang L, Wang J, Xie XQ, et al The autophagy gene *BbATG5*, involved in the formation of the autophagosome, contributes to cell differentiation and growth but is dispensable for pathogenesis in the entomopathogenic fungus *Beauveria bassiana*. Microbiol-SGM. 2013;159:243–252. 10.1099/mic.0.062646-0 23197175

[42] Manafo DB, Colombo MI A novel assay to study autophagy: regulation of autophagosome vacuole size by amino acid deprivation. J Cell Sci. 2001;114(20):3619–3629. 11707514 10.1242/jcs.114.20.3619

[43] Klionsky DJ, Cuervo AM, Seglen PO Methods for monitoring autophagy from yeast to human. Autophagy. 2007;3(3):181–206. 17224625 10.4161/auto.3678

[44] Klionsky DJ, Abdalla FC, Abeliovich H, et al Guidelines for the use and interpretation of assays for monitoring autophagy (3rd edition). Autophagy. 2016;12(1):1–222. 26799652 10.1080/15548627.2015.1100356 PMC4835977

[45] Duan Z, Chen Y, Huang W, et al Linkage of autophagy to fungal development, lipid storage and virulence in *Metarhizium robertsii*. Autophagy. 2013;9(4):538–549. 23380892 10.4161/auto.23575 PMC3627669

[46] Zhang LB, Feng MG Antioxidant enzymes and their contributions to biological control potential of fungal insect pathogens. Appl Microbiol Biotechnol. 2018;102(12):4995–5004. 29704043 10.1007/s00253-018-9033-2

[47] Schmidt AR, Dorfelt H, Perrichot V *Palaeoanellus dimorphus* gen. et sp. nov. (Deuteromycotina): a Cretaceous predatory fungus. Am J Bot. 2008;95(10):1328–1334. 21632336 10.3732/ajb.0800143

[48] Gołębiowski M, Boguś MI, Paszkiewicz M, et al The composition of the cuticular and internal free fatty acids and alcohols from *Lucilia sericata* males and females. Lipids. 2012;47(6):613–622. 22415221 10.1007/s11745-012-3662-5 PMC3357471

[49] Lu YX, Zhang Q, Xu WH Global metabolomic analyses of the hemolymph and brain during the initiation, maintenance, and termination of pupal diapause in the cotton bollworm, *Helicoverpa armigera*. PLoS One. 2014;9(6):e99948. 24926789 10.1371/journal.pone.0099948 PMC4057385

[50] Gould SJ, McCollum D, Spong AP, et al Development of the yeast *Pichia pastoris* as a model organism for a genetic and molecular analysis of peroxisome assembly. Yeast. 1992;8(8):613–628. 1441741 10.1002/yea.320080805

[51] Kiffin R, Bandyopadhyay U, Cuervo AM Oxidative stress and autophagy. Antioxid Redox Signal. 2006;8(1–2):152–162. 16487049 10.1089/ars.2006.8.152

[52] Nitsche BM, Burggraaf-van Welzen AM, Lamers G, et al Autophagy promotes survival in aging submerged cultures of the filamentous fungus *Aspergillus niger*. Appl Microbiol Biotechnol. 2013;97(18):8205–8218. 23700238 10.1007/s00253-013-4971-1

[53] Zhang Y, Qi H, Taylor R, et al The role of autophagy in mitochondria maintenance: characterization of mitochondrial functions in autophagy deficient *S. cerevisiae* strains. Autophagy. 2007;3(4):337–346. 17404498 10.4161/auto.4127

[54] Ashrafi G, Schwarz TL The pathways of mitophagy for quality control and clearance of mitochondria. Cell Death Differ. 2013;20(1):31–42. 22743996 10.1038/cdd.2012.81 PMC3524633

[55] Deng YZ, Qu Z, Naqvi NI Role of macroautophagy in nutrient homeostasis during fungal development and pathogenesis. Cells. 2012;1(3):449–463. 24710485 10.3390/cells1030449 PMC3901100

[56] Kershaw MJ, Talbot NJ Genome-wide functional analysis reveals that infection-associated fungal autophagy is necessary for rice blast disease. Proc Natl Acad Sci USA. 2009;106(37):15967–15972. 19717456 10.1073/pnas.0901477106 PMC2747227

[57] Russell RC, Yuan HX, Guan KL Autophagy regulation by nutrient signaling. Cell Res. 2014;24(1):42–57. 24343578 10.1038/cr.2013.166 PMC3879708

[58] Voigt O, Herzog B, Jakobshagen A, et al bZIP transcription factor *SmJLB1* regulates autophagy-related genes *Smatg8* and *Smatg4* and is required for fruiting-body development and vegetative growth in *Sordaria macrospora*. Fungal Genet Biol. 2013;61(1):50–60. 24095659 10.1016/j.fgb.2013.09.006

[59] Ying SH, Feng MG, Keyhani NO A carbon responsive G-protein coupled receptor modulates broad developmental and genetic networks in the entomopathogenic fungus, *Beauveria bassiana*. Environ Microbiol. 2013;15(11):2902–2921. 23809710 10.1111/1462-2920.12169

[60] Shang Y, Xiao G, Zheng P, et al Divergent and convergent evolution of fungal pathogenicity. Genome Biol Evol. 2016;8(5):1374–1387. 27071652 10.1093/gbe/evw082 PMC4898799

